# Time Averaged Transmitter Power and Exposure to Electromagnetic Fields from Mobile Phone Base Stations

**DOI:** 10.3390/ijerph110808025

**Published:** 2014-08-07

**Authors:** Alfred Bürgi, Damiano Scanferla, Hugo Lehmann

**Affiliations:** 1 ARIAS umwelt.forschung.beratung, Gutenbergstrasse 40B, CH-3011 Bern, Switzerland; 2 Swisscom (Switzerland) Ltd., Innovation, Mobile Access, Ey. 10, CH-3063 Ittigen, Switzerland; E-Mails: damiano.scanferla@swisscom.com (D.S.); hugo.lehmann@swisscom.com (H.L.)

**Keywords:** radio frequency electromagnetic fields, EMF exposure assessment, EMF modeling

## Abstract

Models for exposure assessment of high frequency electromagnetic fields from mobile phone base stations need the technical data of the base stations as input. One of these parameters, the *Equivalent Radiated Power* (ERP), is a time-varying quantity, depending on communication traffic. In order to determine temporal averages of the exposure, corresponding averages of the ERP have to be available. These can be determined as *duty factors,* the ratios of the time-averaged power to the maximum output power according to the transmitter setting. We determine duty factors for UMTS from the data of 37 base stations in the Swisscom network. The UMTS base stations sample contains sites from different regions of Switzerland and also different site types (rural/suburban/urban/hotspot). Averaged over all regions and site types, a UMTS duty factor *F* ≈ 0.32 ± 0.08 for the 24 h-average is obtained, *i.e.*, the average output power corresponds to about a third of the maximum power. We also give duty factors for GSM based on simple approximations and a lower limit for LTE estimated from the base load on the signalling channels.

## 1. Introduction

As there is public concern regarding possible health effects of exposure to radio frequency electromagnetic fields (RF-EMF), the determination of the level of exposure of the general public and/or of selected population samples is of considerable interest. This exposure can be determined either by measurement, e.g., by equipping people with mobile measurement devices, or by modelling.

Mobile measurements with exposimeters can give detailed information on the actual exposure of a person from a large number of sources, however, it is expensive when large study groups are to be considered. Modelling is possible only for those sources which are fixed in space and of which the technical parameters (location, transmission power, frequency and direction *etc.*) are known. Where this is the case, the exposure can be calculated with affordable effort even for extended regions or large groups of study participants.

Models with exposure calculations for selected regions have been presented e.g., by Lehmann *et al.* [1] for regions near Bern and in Zürich, or Bürgi *et al.* [2] for two regions in and near Basel; and model calculations for applications in epidemiological research have been reported by Bürgi *et al.* [3] for the region of Basel and by Beekhuizen *et al.* [4] for the city of Amsterdam. Exposure models for extended regions are also routinely calculated for the region of Central Switzerland [5] and for the cantons of Basel-Stadt and Basel-Landschaft [6], using the same methods as described in [2] and [3]. In addition to the transmitter data, these models also need 3D data on terrain and especially on buildings, as discussed by Beekhuizen *et al.* in [7].

The electric field in front of an antenna, in the simplest approximation, is proportional to the square root of the equivalent radiated power (ERP) divided by the distance from the antenna, and the power flux-density is proportional to the ERP divided by distance squared. However, the power radiated by an antenna for mobile communication is not constant in time, but depends on communication traffic. Typically, low traffic occurs in the late night and early morning, while peak traffic may occur around noon and in the afternoon or evening.

When calculating exposure to RF-EMF fields, a suitable time average has to be applied, e.g., the long-time average over 24 h of each day or a suitable average over selected daytime (or night-time) hours. In this case, a corresponding average of the transmitted power over the period in question is also necessary.

Typically, the average can be characterised by a duty factor, *i.e.*, the ratio of the time averaged power to the maximum operational power as determined by the transmitter setting. In this report, we will review the duty factors that have been estimated by Lehmann *et al.* for GSM, and report new results for duty factors of UMTS that have been determined from actual operational data from a large sample of Swisscom mobile phone base stations. We also estimate a duty factor for LTE.

The application of duty factors in model calculations is straightforward: One simply replaces every occurrence of the ERP in the propagation model equations with the ERP multiplied by the appropriate duty factor to obtain the corresponding time-average of the power flux-density. From this, one can then also calculate the average for the electric field strength.

## 2. Methods

### 2.1. Equivalent Radiated Power, Transceivers and Carriers

In mobile communication networks, base stations are typically sectorized into two or three sectors/cells with different angular directions. Each cell is served by one base station antenna and it is associated to one technology (e.g., GSM, UMTS, LTE) and frequency band. If a base station antenna supports multiple frequency bands, it can be used to serve more cells of different technologies in the same angular direction.

A transmitter/antenna system is comprised of one or several transceiver (transmitter/receiver, TRX) units which feed and receive signals through combiner and duplexer units to and from an antenna. Each TRX unit produces a carrier wave and modulates it with some input signal (voice or data), then amplifies the result. It also amplifies and demodulates received signals.

The combiner combines the outputs from several TRXs, while the splitter splits the received signal into the appropriate inputs of the TRX.

The duplexer separates outgoing and incoming signals to and from the antenna.

The antenna converts the outgoing electric signal in the feeder cable into an electromagnetic wave propagating in space and vice versa for received signals.

The ERP is the sum of the TRX output power (in Watt, W), divided by the total loss in combiner, duplexer and feeder cable, and multiplied by the antenna gain. The antenna gain *G* takes into account the focussing of the radio wave in the forward direction:

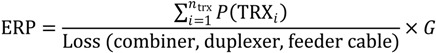
(1)


The number of TRX units (or number of carriers) is designated by the symbol *n*_trx_.

The ERP as defined in (1) is a time-varying quantity. We therefore define the *Operation ERP* (ERP_op_), the ERP at maximum amplification according to the power settings in the TRXs:

ERP_op_ = ERP at maximum amplification according to power setting (2)

Note that this can be (and often is) less than the maximum output power that the TRX can produce, as coverage and cell planning may demand less power or the power is restricted due to exposure limitations, as e.g., in Switzerland [8].

Typical values are *P* = 20 W per TRX, losses of a factor of 2 (3 dB) and an antenna gain of 40 (16 dB), yielding ERP_op_ ≈ 400 W (per carrier). The ERP_op_ determines, among other things, the spatial size of the radio cell.

We define the *duty factor F* as the ratio of a suitably selected time average 〈ERP〉 and ERP_op_:

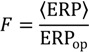
(3)


We will use three different duty factors *F*_24_, *F*_day_ and *F*_night_, where the averages in 〈*ERP*〉 are over the whole day (*F*_24_), the hours from 06:00 to 22:00 (*F*_day_) and 22:00 to 06:00 (*F*_night_), respectively. Note that the “*day”* defined in this way is twice as long as the “*night”,* and the day/night definition has been taken the same as in the Swiss noise abatement regulation [9]. The duty factors will be numbers between 0 and 1.

### 2.2. GSM

In GSM the signal of each carrier is divided into data frames of 4.6 ms, with each frame further divided into 8 timeslots. Each timeslot can be attributed to a different user. A GSM base station can have one or several TRX.

The first TRX is used for the *Broadcast Common Control Channel* (BCCH). The BCCH is always transmitted at full power in all timeslots. The remaining TRX are used as *Traffic Channels* (TCH). In a TCH, a signal is present only in those timeslots that are actually used for communication. In addition, the TCH can use adaptive power control, *i.e.*, the power is reduced to a lower level when the connection is good. Consequently, the time averaged power on a TCH is considerably less than the maximum.

A frequency channel in GSM has a width of 200 kHz. The BCCH always uses a single frequency channel. A TCH can use more than one frequency channel when “*frequency hopping”* is used, in order to improve the communication quality. With frequency hopping, the frequency channel of a TCH can be changed after every data frame. For this reason, the number of frequency channels allocated to a GSM cell can be larger than the number of carriers *n*_trx_.

### 2.3. UMTS

#### 2.3.1. General Considerations

UMTS uses a bandwidth of 5 MHz per frequency block. The 2100 MHz band can accommodate 12 such blocks (for uplink and downlink each). With several network operators sharing the total bandwidth, only a few frequency blocks will be available per operator.

UMTS uses code division multiple access (CDMA). All base stations use the same frequency block(s). Each signal is spread over the entire block width, and multiple users and communication channels are distinguished by different spreading codes. Spreading codes are mutually orthogonal, *i.e.*, they have zero cross-correlation when they are perfectly synchronised.

UMTS base stations use a “*Common Pilot Channel”* (CPICH) which transmits at constant power. The CPICH is typically set to about 10% of the ERP_op_. Other (secondary) signalling channels may add another 5% to 10% to the constant load.

The power of the communication channels depends on the communication traffic. A very effective adaptive power control is used to minimize power in communication channels, in order to reduce interference between different users.

A UMTS cell may use several transceiver units (TRX). Each TRX (or carrier) uses a different frequency block, and multiple TRX of the same cell operate (quasi) independently, *i.e.*, each TRX has its CPICH and other signalling channels. For UMTS, the number of carriers *n*_trx_ is equal to the number of frequency channels (*i.e.*, frequency blocks) used.

Due to the presence of the signalling channels (CPICH and others) the lower limit for the duty factors can be expected to be in the range 0.15 to 0.20. The duty factor can also be expected to be well below the maximum of 1, as the network must be designed to handle intermittent periods of high traffic. Should all channels always transmit at full power, this would mean that the users near the edge of the cell would suffer from degraded communication quality due to interference, or even be disconnected from the cell, and new users would not be able to access the network. Network operators therefore have a high motivation to redesign their network under such conditions, either by shrinking the cell size (*i.e.*, the power) and adding additional cells, or by adding additional carriers to a cell with heavy load. However, they will also tend not to install more carriers and/or power than needed due to financial considerations. The resulting duty factors are therefore influenced by technical constraints (e.g., base load from signalling channels) on the one hand and by a trade-off between network capacity and cost on the other hand. As the principles of network capacity planning are similar for different countries and providers, one can expect similar duty factors in other networks as well. Exceptions to this might be small operators with limited investment capability and/or lower requirements for user throughput, who might accept a higher load on their network, e.g., in developing countries.

#### 2.3.2. UMTS Power Monitoring

Data of the UMTS output power was obtained for 37 UMTS base stations of Swisscom, comprising 97 cells. The data are based on 15-min averages of the ERP recorded during one month from 10 June 2013 to 10 July 2013.

The base stations were selected from different regions in Switzerland (categorized as Central, North, South and West) and for different types of sites (rural, suburban, urban and hotspot). Hotspots are located near heavily frequented locations, e.g., airports or train stations. All cells are outdoor macrocells, *i.e.*, having cell radii ranging from several hundred meters to several kilometers.

**Figure 1:**
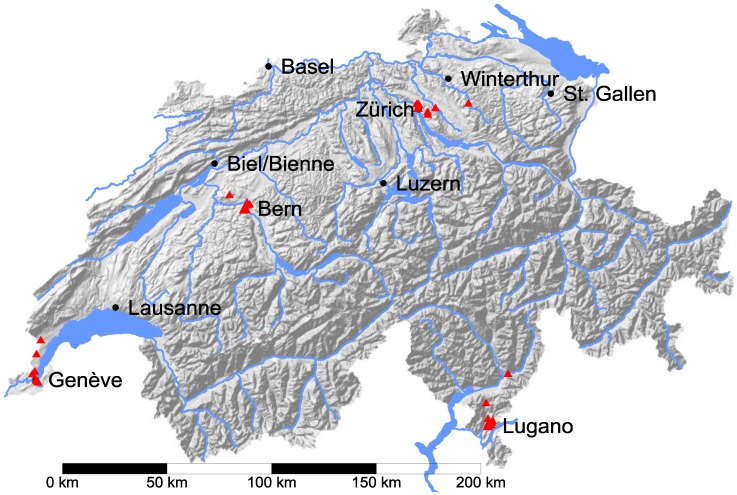
Location of the UMTS base station sample (red triangles) on the map of Switzerland. As the stations in the sample are strongly clustered, it is difficult to distinguish individual sites at this map scale. Background map data © 2014 BfS ThemaKart (vector data) and Swisstopo (DHM25/200).

The location of the sample of UMTS base stations is shown in Figure 1. One can clearly distinguish the groups in the four regions. The four regions represent different cultural and language regions (North and Centre (near Zürich and Bern): German, West (near Geneva): French, South (near Lugano): Italian) and also different topographical conditions, which might be reflected in the use of mobile phones and hence in the duty factors.

Depending on the grouping, the sample consists of between eight and ten base stations per region and between seven and eleven base stations per site type. The purpose of the grouping is to find out if there are significant differences between site types or regions. As we have only data from one country, stratification between different regions is so far the only possibility to determine possible spatial variations in the duty factors.

In order to find a possible dependence of the UMTS duty factor on base station power and/or the number of carriers, we have calculated the Pearson correlation coefficient between *F*_24_ and the two quantities ERP_op_ and *n*_trx_.

### 2.4. LTE

LTE, the *Long Term Evolution* standard, defines the evolutionary steps in mobile communication networks after UMTS. LTE can be used in a variety of frequency bands (e.g., the 800, 900, 1800, 2100, 2600 MHz bands), and it can be configured for a variety of bandwidths (1.4, 3, 5, 10, 15 and 20 MHz).

LTE uses *Orthogonal Frequency Division Multiple Access* (OFDMA) for the downlink. The basic substructure in time is a subframe of 1 ms duration. A subframe is in turn divided into two *slots*, and each slot is 7 *symbols* long*.* The basic data structure in LTE is a *Resource Block* (RB). A resource block consists of 12 subcarriers (15 kHz wide each) and has a length of 1 slot. A *resource element* (RE) is one subcarrier wide in frequency and one symbol long in time. It can contain 2, 4 or 6 bits of information, depending on the modulation.

The *Cell specific Reference Signal* (CRS) is a signalling channel transmitted at constant power. The CRS uses 4 RE from every RB, *i.e.*, it uses a fraction of 4/84 = 4.8% of all RE. The second most important contribution to the baseline power are the invariably present primary and secondary synchronisation (P-SS and S-SS) channels as well as the *physical broadcast channel* (PBCH). P-SS and S-SS expand over the 62 RE in the frequency centre of the signal and are emitted each 5 ms. The PBCH appears also in the centre of the signal once each 10 ms and fills up the 72 RE of the six central RB. The *Primary Downlink Control Channel* (PDCCH) can be adjusted according to the particular system configuration, traffic scenarios and channel conditions. It uses a part of the one to three first symbols of every subframe, but its contribution can vary depending on the configuration.

The transmission power per RE (or *Energy per resource element*, EPRE) is not necessarily the same for all transmission channels. Depending on the target trade-off between coverage and capacity, the operator can assign higher power to the data RE than CRS RE (maximising capacity), lower power to data RE than CRS RE (maximising coverage) or same power for both. Typical offsets between the EPRE of different channels could be of the order of ±3 dB.

RE that are not used for the signalling channels can be used to transmit user data. RE that contain neither signalling nor user data are empty and do not contribute to the output power. Therefore the transmitter power will depend on the instantaneous sector RE utilisation rate.

We have no LTE power measurements available so far. However, a lower limit to the duty factor can be derived by counting the RE in the signalling channels and relating their power to the total power available for all RE.

## 3. Results and Discussion

### 3.1. Duty Factors for GSM

Traffic-dependent GSM transmitter power is not a continuously monitored quantity and would have to be deduced from other load-dependent parameters. Therefore, one has to rely on approximations for the GSM duty factor. Such an approximation has been given by Lehmann *et al.* [1] for the 24 h average power. It is based on the fact that the first carrier (the BCCH) is always on full power, while the contribution of additional carriers decreases with increasing *n*_trx_. The approximation for the traffic channels is an educated guess based on empirical data from a number of Swisscom base stations.

A very similar sequence of duty factors as in [1] can be derived by the approximation that the first carrier (the BCCH) contributes 100%, the second carrier 20%, and all further ones 10% of the power per carrier. This can be expressed by:

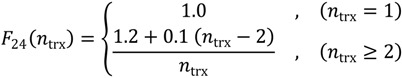
(4)
and gives the duty factors *F*_24_ in the third column of Table 1 (the factors are identical to those of [1] for *n*_trx_ ≤ 4). Assuming further that during the night hours (22–06 h) only the BCCH is active, the night-time duty factors scale as:

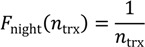
(5)


From these and *F*_24_ it is the easy to derive duty factors for the daytime hours (*F*_day_), which are also given in Table 1.

**Table 1:** Estimated duty factors for GSM cells as a function of the number of carriers in the cell.

Number of carriers *n*_trx_	*F*_24_, Lehmann *et al.* [1]	F_24_ this paper	*F*_day_	*F*_night_
1	1.00	1.00	1.00	1.00
2	0.60	0.60	0.65	0.5
3	0.43	0.43	0.48	0.33
4	0.35	0.35	0.40	0.25
5	0.28	0.30	0.35	0.20
6	0.25	0.27	0.32	0.17

### 3.2. Duty Factors for UMTS

The basis for the determination of the UMTS duty factors were the recorded 15-min averages of the actual ERP related to ERP_op_, provided by Swisscom, as described in Section 2.3.2. In addition to *F*_24_, *F*_day_ and *F*_night_, also the minimum and maximum ratios were determined:

- *F_min_*: The minimum of the 15-min ERP averages divided by ERP_op_.
- *F_max_*: The maximum of the 15-min ERP averages divided by ERP_op_.

The duty factors determined in this way were then grouped according to regions and cell types, and averages as well as standard deviations of the duty factors for these groups were calculated. They are denominated as:

- 〈*F*_24_〉 *: F*_24_ averaged over the cells of a group

and analogous for 〈*F*_day_〉, 〈*F*_night_〉, 〈*F*_min_〉 and 〈*F*_max_〉

**Figure 2:**
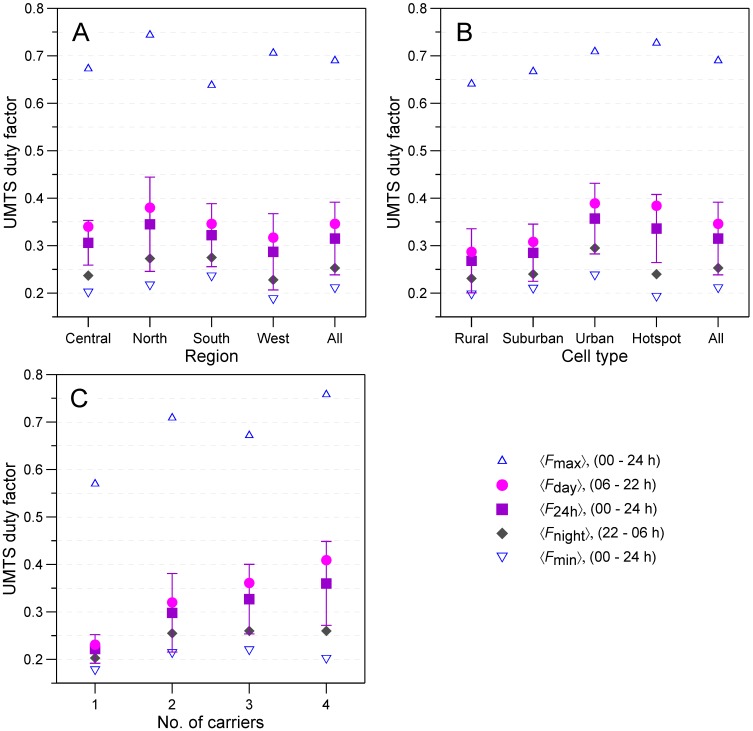
(**A**) (upper left): ERP duty factors, averaged over cells by region. (**B**) (upper right): ERP duty factors, averaged over cells by cell type. (**C**) (lower left): ERP duty factors, averaged over cells by number of carriers. Also shown is the standard deviation for *F*_24_.

The average ratios for the different regions are shown in Figure 2A. The numerical values of all the data in the figure are given in tabular form in the supplementary information file.

The region-averaged 24h-duty factors are in the range 0.29–0.34, while the overall average over all the cells is 〈*F*_24_〉 = 0.32 ± 0.08 (sample average ± standard deviation). The values for the median (0.31) and interquartile range (0.09) of *F*_24_ are very similar to the mean and standard deviation (see Table S1 in the supplementary information file). Figure 2A also shows the standard deviation of the groups. It is visible that the variation within the groups is larger than the differences between groups, *i.e.*, there is no significant difference in the 24h-duty factor among the regions.

The day-time duty factors *F*_day_ are somewhat higher than the 24 h-values: they range between 0.32 and 0.38, with an ensemble average of 〈*F*_day_〉 = 0.35 ± 0.09, while the duty factors for the night hours are distinctly lower (0.23–0.28), with an overall average of 〈*F*_night_〉 = 0.25 ± 0.06.

The group-averaged minimum values range between 0.19 and 0.23. This corresponds well with a base load of approximately 10% from the CPICH plus a similar amount from other signalling channels. The region-averaged 15-min maxima are as high as 0.64–0.74, which is considerably higher than the time averages.

The average of 〈*F*_24_〉 = 0.32 corresponds well with the value 0.25 assumed by Lehmann *et al.* for 2004, considering that UMTS traffic has increased considerably in the last ten years.

Figure 2B gives the group-averaged duty factors in the same format as Figure 2A, but averaged over cell types. Here also, the variation within the groups is still comparable to the differences between groups, but a clear increase of the duty factors from rural and suburban to urban and hotspot is visible.

It is interesting to note that all three factors, *F*_24_, *F*_day_ and *F*_night_, are higher for “urban” than for “hotspot”. This could possibly be explained by the fact that near "hotspots" the base station density is higher. Thus users would be closer to the base station and less power would be needed. Consequently, the duty factor would be less than for “urban”, when the transmission power and volume are similar. In addition, more indoor small cells can be found near “hotspots”, offloading some of the traffic from the “hotspot” and reducing its duty factor. The most extreme maximum and minimum values, however, occur for “hotspot”. This implies that a hotspot is “hot” during certain peak hours only, but relatively quiet at other times of day when compared to the other cell types.

In order to find systematic influences, we also looked at correlations between the variables. The first correlation we examined was between ERP_op_ and the duty factor *F*_24_ where we found a correlation coefficient *ρ* = 0.045, *i.e.*, practically no correlation at all, showing that high or low traffic may occur both in big and small cells.

A more significant correlation coefficient, *ρ* = 0.46 was found between the number of carriers *n*_trx_ and the duty factor *F*_24_ meaning that the duty factors for UMTS depend on the installed number of carriers in a way similar to GSM. The cells in the sample use between one and four carriers.

Figure 2C shows the duty factors as a function of the number of carriers *n*_trx_. Clearly, the duty factors increase with the number of installed carriers, ranging from *F*_24_ = 0.22 (*n*_trx_ = 1) to 0.36 (*n*_trx_ = 4). The dependence on *n*_trx_ for UMTS is different from that for GSM. Duty factors for GSM decrease with *n*_trx_, while duty factors for UMTS increase.

**Figure 3:**
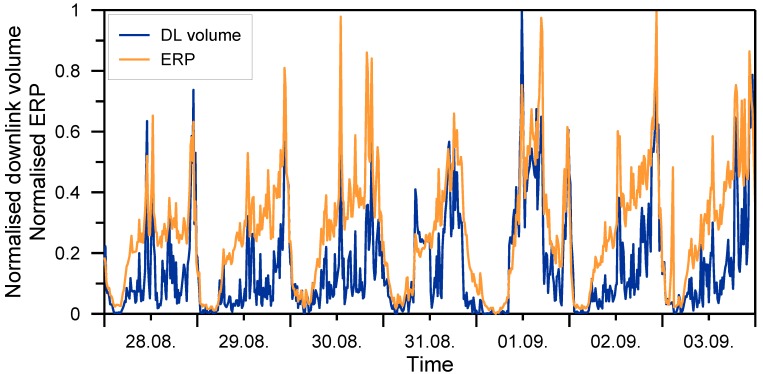
Comparison of the normalised downlink data volume and the normalised ERP of a UMTS cell for one week of data.

The relation between downlink traffic volume and ERP is illustrated in Figure 3. Displayed are the 15-min averages of the downlink data volume (in MB) and the ERP (in W), both normalized to the interval from 0 to 1. The data are for one cell, covering one week in August/September 2013. The figure clearly shows how similar the peaks are. Calculating the correlation between the values of ERP and downlink data volume gives a Pearson correlation coefficient *ρ* = 0.82, *i.e.*, a very high correlation.

All UMTS measurements were made during one month in summer. Clearly, the traffic (voice and data) and hence the duty factor vary during the course of the year. Typically, they are lower during Christmas, Easter and summer. The analysis of the traffic of the 37 base stations showed that the volume started to decrease around the 5th–10th of July due to the start of the summer holidays, reached a minimum around the end of July and then increased back until the middle of August. So, although the last days of the measurements are affected by the holiday season, most of the period is not.

### 3.3. Duty Factors for LTE

In Switzerland, the first LTE base stations have been put into service at the end of 2012. So far, they mostly operate in the 1800 MHz band. Unfortunately, it was not yet possible to derive LTE duty factors from actual data, as there are no counters for instantaneous transmission power (as yet). On the other hand, as the LTE networks are still evolving fast, both in terms of base station coverage and available user equipment, duty factors derived from data at this point in time might be restricted in validity to the current transient state of the network.

Similarly to UMTS, LTE has a power base load produced by the signalling channels. The main channels contributing to this are the cell specific reference signal CRS, the synchronisation channels (P-SS and S-SS) as well as the physical broadcast channel PBCH. By counting resource elements used by these channels, one obtains a minimum duty factor of 5.3% for a signal bandwidth of 20 MHz. For the smallest LTE bandwidth (1.4 MHz) the minimum duty factor increases to 10%. Furthermore, the settings of the PDCCH may considerably influence the base load. For a 10 MHz bandwidth, counting CRS, P-SS, S-SS, PBCH and assuming a PDCCH of one symbol in each subframe, all at equal power per RE, one obtains a minimum duty factor of 13%.

It is difficult to predict LTE duty factors at this stage. As in UMTS, the time averages can be expected to be well below 100%, so that there is a margin to handle traffic at peak times. As a rough guess, an LTE duty factor might be estimated at this time (analogous to the UMTS duty factor *F* ≈ 0.25 in [1]).

## 4. Conclusions

We have presented duty factors (*i.e.*, time averaged ERP divided by operational ERP power setting) for GSM, UMTS and LTE. Such factors are necessary for calculating average exposure in numerical models. The factors can also differentiate between day, night and all-day exposure.

For GSM, the duty factors are essentially those of Lehmann *et al.* [1], with an extensions to values for day and night.

For UMTS, duty factors have been derived from recorded transmitter power data, from 97 cells of 37 base stations of Swisscom. The sample was taken such as to cover a range of different site types (rural, suburban, urban, hotspot) and regions of Switzerland. For the ensemble of cells, we found average duty factors of

*F*_day_ = 0.35 ± 0.09 *F*_night_ = 0.25 ± 0.06 *F*_24_ = 0.32 ± 0.08 (6)

for the daytime, night-time and whole-day averages, respectively. The indicated variability is the standard deviation (1*σ*) of the sample.

From the variation indicated by the standard deviation, it is clear that the derived duty factors are valid in a statistical sense, and that there can be a considerable variation (about ± 25%) between cells.

We also found a dependence of the UMTS duty factors on the number of carriers, such that the cells with higher numbers of carriers show higher duty factors. This can be attributed to the necessity to install additional carriers in cells with high traffic. However, this relation may be of limited validity, especially the low duty factors for cells with a single carrier. These might reflect the fact that most UMTS cells in the present Swisscom network are now equipped with two or more carriers, leaving only the cells with very low traffic having just a single carrier. The low duty factors for *n*_trx_ = 1 might not be applicable to networks of other operators and/or to the past. For example, the competitors of Swisscom have roughly a third of its market share (and traffic) each, so most of their UMTS cells can be expected to use one or two carriers, showing corresponding duty factors.

All UMTS measurements were made during one month in summer. However, only the last days of the period were affected by a decrease in traffic due to the start of the holiday season. As typically the yearly variation of the traffic is much smaller than the daily one, the derived duty factors should be valid for the whole year.

For LTE, no measurements were available so far, and only a lower limit to the base load from the signalling channels could be derived.

The duty factors given for GSM were estimated from experience with the Swisscom GSM network, and the UMTS duty factors reported here are based on measurements in the Swisscom UMTS network. From the successful validation of models given in references [1] to [6], and especially [4] (in the Netherlands), where similar duty factors to those reported here were used, it can be concluded that these values in general also apply to other networks. Even though we have only considered data from one country, the absence of differences in the duty factors between the regions, with different cultures/languages and topography, also supports this conclusion. As explained in Section 2.3.1, the duty factors are the result of a trade-off between cost and capacity, and as all network systems are operated in a similar manner, similar duty factors should also apply elsewhere. We have not considered small cells in our study, but by the same reasoning one can argue that their duty factors should also be similar to those of larger cells. However, it would be very interesting to have data from networks of other operators and in other countries in order to make a comparison.

Finally, it would also be interesting to have actual measured transmitter power data for LTE and to a lesser extent for GSM, as well as measurements for outdoor small cells. Repeated UMTS measure­ments in future years could also indicate how these duty factors evolve with time.

## Supplementary Files

Supplementary File 1
