# Supplementary material for: Time Averaged Transmitter Power and Exposure to Electromagnetic Fields from Mobile Phone Base Stations
Source: Int J Environ Res Public Health. 2014 Aug 7;11(8):8025–37. doi: 10.3390/ijerph110808025 (PMC4143847; doi:10.3390/ijerph110808025)
Supplement: Supplementary File 1 [file ijerph-11-08025-s001.pdf]

Time Averaged Transmitter Power and Exposure to Electromagnetic Fields from Mobile Phone Base Stations

Table S1. UMTS duty factors as a function of the region, displayed as average ± standard deviation of the group. The row labelled "All" shows the average factors for the whole dataset. The median and the quartiles Q_{25} and Q_{75} are also given in the last three rows. The statistics are based on the respective sample(s) of cells.

**Region**	**# cells**	F_{min}	F_{night}	F_{24}	F_{day}	F_{max}
Central	27	0.20 ± 0.04	0.24 ± 0.04	0.31 ± 0.05	0.34 ± 0.05	0.67 ± 0.15
North	24	0.22 ± 0.05	0.27 ± 0.08	0.34 ± 0.10	0.38 ± 0.12	0.74 ± 0.17
South	24	0.23 ± 0.05	0.28 ± 0.05	0.32 ± 0.07	0.35 ± 0.07	0.64 ± 0.14
West	22	0.19 ± 0.04	0.23 ± 0.06	0.29 ± 0.08	0.32 ± 0.10	0.71 ± 0.22
All	97	0.21 ± 0.05	0.25 ± 0.06	0.32 ± 0.08	0.35 ± 0.09	0.69 ± 0.17
Median	97	0.21	0.25	0.31	0.34	0.68
Quartile Q_{25}	97	0.17	0.20	0.26	0.28	0.59
Quartile Q_{75}	97	0.23	0.28	0.35	0.39	0.79

Table S2. UMTS duty factors as a function of the cell type, displayed as average ± standard deviation of the group. The last row shows the average factors for the whole dataset.

**Cell Type**	**# cells**	F_{min}	F_{night}	F_{24}	F_{day}	F_{max}
Rural	18	0.20 ± 0.04	0.23 ± 0.05	0.27 ± 0.07	0.29 ± 0.08	0.64 ± 0.20
Suburban	27	0.21 ± 0.04	0.24 ± 0.05	0.28 ± 0.06	0.31 ± 0.07	0.67 ± 0.21
Urban	27	0.24 ± 0.04	0.30 ± 0.06	0.36 ± 0.07	0.39 ± 0.08	0.71 ± 0.14
Hotspot	25	0.19 ± 0.05	0.24 ± 0.06	0.34 ± 0.07	0.38 ± 0.08	0.73 ± 0.15
All	97	0.21 ± 0.05	0.25 ± 0.06	0.32 ± 0.08	0.35 ± 0.09	0.69 ± 0.17

Table S3. UMTS duty factors as a function of the number of carriers, displayed as average ± standard deviation of the group. The average number of carriers in the dataset is 2.7. The last row shows the average factors for the whole dataset.

n_{trx}	# cells	F_{min}	F_{night}	F_{24}	F_{day}	F_{max}
1	9	0.18 ± 0.02	0.20 ± 0.03	0.22 ± 0.03	0.23 ± 0.03	0.57 ± 0.26
2	28	0.21 ± 0.05	0.26 ± 0.06	0.30 ± 0.07	0.32 ± 0.08	0.71 ± 0.18
3	42	0.22 ± 0.04	0.26 ± 0.05	0.33 ± 0.06	0.36 ± 0.07	0.67 ± 0.16
4	18	0.20 ± 0.06	0.26 ± 0.08	0.36 ± 0.08	0.41 ± 0.09	0.76 ± 0.14
2.7 (average)	97 (all)	0.21 ± 0.05	0.25 ± 0.06	0.32 ± 0.08	0.35 ± 0.09	0.69 ± 0.17

© 2014 by the authors; licensee MDPI, Basel, Switzerland. This article is an open access article distributed under the terms and conditions of the Creative Commons Attribution license (http://creativecommons.org/licenses/by/3.0/).
